# Supplementary material for: Antipsychotic-induced epigenomic reorganization in frontal cortex of individuals with schizophrenia
Source: eLife. 2024 Apr 22;12:RP92393. doi: 10.7554/eLife.92393 (PMC11034945; doi:10.7554/eLife.92393)
Supplement: Supplementary file 7. [file elife-92393-supp7.docx]

**Supplementary File 7. Enrichment of various GWAS traits in differential enhancers and promoters.** Significance is presented as -log(p values) using LD score regression (ldsc v1.0.1) using genetic loci previously associated with schizophrenia risk (Trubetskoy et al., 2022) as well as other brain and non-brain related traits (Finucane et al., 2018). High values are in red and low values are in blue.
